# Nghozi na Nxungeto Ku valanga nhlamuselo hi tihlo ra tindzimi ta Afrika (Dzonga): Mhaka ya Xitsonga eka mbulavulo wa khombo

**DOI:** 10.4102/jamba.v17i1.1901

**Published:** 2025-05-09

**Authors:** Norman Mathebula, Morris T. Babane, Jabulani C. Makhubele, Kingsley K. Ayisi, Wiseman N. Mathebula, Prudence Mafa, Josephine M. Letsoalo, Mzamani S. Khosa, Meshack O. Shilenge

**Affiliations:** 1 Centre for Global Change, Faculty of Science and Agriculture, University of Limpopo, Polokwane, South Africa; 2 Department of African Languages, Faculty of Humanities, Social Sciences and Education, University of Venda, Thohoyandou, South Africa; 3 Directorate of Research and Innovation, University of Venda, Thohoyandou, South Africa; 4 Department of Social Work, Faculty of Humanities, University of Limpopo, Polokwane, South Africa; 5 Department of Geography and Environmental Studies, Faculty of Science and Agriculture, University of Limpopo, Polokwane, South Africa; 6 Department of English, Media Studies and Linguistics, Faculty of Humanities, Social Sciences and Education, University of Venda, Thohoyandou, South Africa; 7 Department of Social Development, Mopani District, Giyani, South Africa

**Keywords:** disaster, risk, crisis, emergency, resilience, vulnerability

## Abstract

**Disaster and Risk! Exploring meaning through the lens of (South) African languages: A case of Xitsonga language in disaster communication.** Even though there is a difference between the disaster management-related terms disaster itself, crisis, and emergency, they are closely interconnected, interdependent, and overlap significantly. With a blurred line between the definitions of these terms, the mainstream literature uses the terms disaster, crisis, and emergency interchangeably and in combination such as ‘disaster crisis management’ and ‘crisis and emergency management’. The aim of this article is to explore the various meanings of ‘disaster’ within the cultural, communication, and language context of the Xitsonga speakers. A qualitative conceptual content analysis has been carried out to establish the differences and similarities between the disaster terms based on interviews with the Xitsonga speaker. The analysis reveals that all are associated with the sudden nature of the event and the damage caused in the process. Furthermore, the study found that there are many common features associated with the terms leading to them being used interchangeably up to a certain extent. There were differences regarding some terms such as ‘danger’ based on the selected two regions of the Xitsonga population.

**Contribution:**

The study concludes that both crisis and emergency would lead to disaster if the event were neglected or mismanaged. The study is significant because it highlights the gaps existing among the Xitsonga speakers in the use of Xitsonga concepts and definitions.

## Manghenelo

Ririmi i xitirhisiwa xa nkoka xo burisana na ku humelerisa miehleketo, mavonelo, na ku twisisa mayelana na mhaka yo karhi. Ri tlhela ri tirhisiwa ku hlamusela, ku komba, na ku xaxameta vanhu, swiharhi, migingiriko, na mikarhi. Na hi nkarhi wa timhangu, ku nga va ta ntumbuluko kumbe to endliwa yi munhu, ririmi ri tirhisiwa ku nyika tinhlamuselo eka swiyimo swoleswo. Ku tiya ka mhaka leyi ku seketeriwa hi Bodenreider et al. (2019), va kombisile leswaku ririmi ri na nkoka eka mbulavulo wa swa timhangu. Hi ririmi, tanihi laha swi boxiweke hi McKee et al. (2012), mahungu ya mikarhi na mikarhi ya ni nkoka eka vutomi bya vaaki lava khumbekaka hi nkarhi wa timhangu. Ntirho wa ririmi eka ku hangalasa switiviso swa timhangu na mahungu yo ntshuxeka wu na nkoka eka angulo wa nxungeto, mbulavulo wa nxungeto, na ku tilulamisela (Gerber, Zhang & Xiang 2018; Peguero 2006; Taibah & Andrew 2014). Ku na ku amukela eka vulavisisi bya mhangu ka leswaku, nahambi swi komba ku lunghiseka loku hlamarisaka, vavulavuri va tindzimi letitsongo va le ka nxungeto wa timhangu ku tlula lavan’wana, naswona leswi swi vangiwa hi mpfumaleko wa ndzingano lowu veke kona (eds. Bates & Swan 2007; Klinenberg 2002). Ku ka ku nga hangalasiwi mahungu ya swa timhangu hi ririmi ra xikaya swi nga va mhaka ya rihanyu na rifu (O’Brien & Federici 2019). Vumbhoni bya ntiyiso kusuka eka nxungeto, ririmi, na tidyondzo ta mbulavulo byi komba leswaku swikanganyisi swa ririmi swi nga vangela swiyimo swa nxungetovutomi eka lava nga ringanelangiki eka tindzimi ta vuhlanganisi leti tirhisiwaka eka mimbulavulo ya khombo erixakeni. Leswi swi nga fikelerisa ntwanano wu nga fikeleriwangi ku twisisa swilo swo fana na xiphiqo xa mhangu, nghozi yo tika, switiviso swo lemukisa, na mahungu yo balekela vuhlayiseki (Aguirre 1988). Swingaleswi, eka swiyimo swa makhombo, mahungumbulavulo wa ririmi leswi ku burisaniwaka kumbe swi nga burisaniwiki hi ndlela yihi – swi nga ha vangela rifu kumbe ku hanya. Swikanganyisi swa ririmi leswi hanyaka swi nga ha nyanyiseka loko mahungu ya xihatla ya burisaniwa hi ririmi kumbe tindzimi ta vuhlanganisi. Valavisisi va swa tidyondzo ta *khombo* va kandziyisile no hlamusela timhaka to hambana to fambelana na tindzimi eka timhangu leti endlekeke eka nkarhi lowu nga hundza (Benavides & Arlikatti 2010; Santos-Hernández & Morruw 2013). Mpfumaleko wa vutivi bya tindzimi na ndhavuko eka mhangu na swiphiqo swa mbulavulo swi nga ha vangela switandzhaku swo chavisa leswi nga papalatiwaka. Ku fikelela mbulavulo wo hetiseka no twisiseka hi ku tirhisa ririmi rin’we (kumbe ririmi ra vuhlanganisi), ku fana na ra Xinghezi eka xifundzhakulu, a swi koteki handle ko tekela enhlokweni ririmi ra le kaya kumbe vuhundzuluxi bya rona (Federici 2020). Mbulavulo wa mhangu eka levhele ya xifundzakulu, xik: Hluvukiso wa Matiko ya Dzonga wa Afrika (SADC), ma fanele ma kongomisa eka ririmi ra le kaya hi tiko eka xifundzhakulu ku engetela nghenelelo wa matiko hinkwawo (Nemakonde et al. 2021). Ku twisisa rito *mhangu* na matheme mo fana na *swaxihatla, nghozi, xiphiqo, nkayakayo na nkavuhlayiseki* eka xiyimo xa tindzimi ta xikaya swi nga yisa eka maqhinga ya kahle yo tala, ku nga ha va ku yimisa kumbe ku hunguta hangalako wa timhangu ta makhombo eka rixaka na mabindzu, na ku hangalaka na ntikelo wa swona eka vanhu na mabindzu. Yan’wana ya matheme lawa, xik: *khombo, nkayakayo,* na *swaxihatla* ma tala ku tirhisiwa hi ncincano, hambileswi ma nga na tinhlamuselo to hambana (Lighthouse Readiness Group 2015). Ku pfuneta ku antswisa ku twisisa swoleswo, ku ringetiwile ku ntlhatlha marito *khombo, nkayakayo,* na *nkavuhlayiseki* hi endlelo ra tihlo ra xintu hi ku tirhisa vavulavuri va Xitsonga; ku nga van’wana va tindzimi ta vavulavuri lavatsongo eAfrika-Dzonga. Kahlekahle, atikili leyi yi vumbiwile hi ndlela leyi: Xo sungula, yi andlala maendlelo lama tirhisiweke, ku landzela tinhlamuselo ta khombo kusuka eka matsalwa na ku huma eka nhlokohliso wa swivutiso lowu nga endliwa na vavulavuri va Xitsonga. Dyondzo leyi i nkatsakanyo wa mpfuxeto wa matsalwa/mahungu mo hlela njhekanjhekisano na njhekisanovuyeto bya xisisiteme, vuhleri bya khombo, nkayakayo, na swaxihatla ku fikela namuntlha na ku tlakusa vutivi lebyi nga kona eka nkarhi wa sweswi eka tlhelo ra dyondzo leyi.

## Xatatimente xa xiphiqo

Hakanyingi, ku onha ka mhangu a hi kun’we hambi ku ri eka rixaka rin’we hi ku landza ku hambana ka nkavuhlayiseki bya rixaka loku fambelanaka na nkucetelo wa swa vanhu swo fana na rimbewu na ririmi (Cutter, Boruff & Shirley 2003). Hikuya hi Campbell, Kamryn and Mary (2020), mahungu hi switsundzuxo ma na nkoka eka miganga leyo swela, ku katsa ni vanhu lava kumaka miholo leyitsongo, na lava nga na mitlhontlho ya ririmi. Hambiswiritano, mitlawa ya vanhu lava tshikeleriweke a swi talangi va katsiwa hi xihatla loko ku kunguhatiwa swaxihatla. Leswi swi vangiwa hikwalaho ka ku va mahungu mayelana na switsundzuxo swa makhombo ma hangalasiwaka hi tindzimi ta vuhlanganisi. Kun’wana na kun’wana emisaveni, ku fana na laha Afrika-Dzonga, ririmi ra ntolovelo ro vika timhangu/mixungeto i ra Xinghezi. Leswi swi siya vavulavuri va tindzimi leti nga riki ta vuhlanganisi va va eka nxungeto wa timhangu hikuva a va nga ti baleki. Nkoka wo katsa tindzimi to swela wu kombisiwile eka mimbulavulo ya timhangu ku ringana malembe yo tala hi ku landza vutivi bya khale (Gomez & Hart 2013). Hambiswiritano, hambiloko ku katsiwa ka tindziminyingi na/kumbe ku tirhisa tindzimixikaya swi pfumeleriwile, leswi swi endliwile ngopfu eka matiko yo fana na Japani. EAfrika-Dzonga, Xinghezi xi ya emahlweni ku va xi ri xona lexi rhangeke eka mbulavulo wa timhangu ta makhombo hambiloko tindzimi hinkwato ti tekiwa tanihi tindzimi ta ximfumo. Hi ku tlhandlekela, ku na nkayivelo wa milavisiso leyi kongomanaka na mitirho ya mimbulavulo hi makhombo leyi tirhisaka Xitsonga tanihi rin’wana ra tindzimi leti tshikeleriweke kumbe ku tsan’wiwa eAfrika-Dzonga.

## Swikongomelo

Dyondzo leyi yi lavisisiwile hi ku kongomisa eka swikongomelo leswi landzelaka:

- Ku kambela tinhlamuselo to hambana ta matheme yo fambelana na mhangu eka Xitsonga
- Ku kuma matirhiselo ya matheme yo fambelana na mhangu eka mbulavulo wa makhombo hi vavulavuri va Xitsonga
- Ku kumisisa ndlela leyi matheme lama ma hambanaka no yelanisa xiswona eka vukhongeri byo hambanahambana bya Afrika-Dzonga.
- Ku kambela nhlohlotelo lowu tindzimi tin’wana ta Afrika-Dzonga ti nga na wona eka matirhiselo ya matheme yo fambelana na khombo.

## Maendlelo na andlalo swa ndzavisiso

Xivumbeko xa dyondzo leyi xi hlohloterile leswaku endlelo ra nkoka ku va rona ri tirhisiwaka eka yona. Eka endlelo ra nkoka, ku na tithekiniki to hambana ta mahlengeletelo ya mahungu. Tithekiniki to fana na nxopaxopo wa matsalwa, ku valanga, na nhlokohliso wa swivutiso. Thekiniki leyi tirhisiweke eka atikili leyi hileya nxopaxopo wa matsalwa. Mahungu ya matsalwa ya pfunetiwile hi nhlokohliso wa swivutiso lowu endliweke na vavulavuri va Xitsonga kusuka emigangeni yo hambana laha Xitsonga xi vulavuriwaka kona. Xiphemunyana xo karhi xa mahungu xi hlengeletiwile kusuka eka Dikixinari ya Tsonga/English. Vanyiki va mahungu va hlawuriwile kusuka eka vatsari va Xitsonga eswifundzheni laha vavulavuri va Xitsonga va kumekaka kona. Endzhaku ka ku nyika nhlamuselo ya theme kusuka eka nhlokohliso wa swivutiso, theme rin’wana na rin’wana ri kamberiwile hi ku landza nhlohlotelo wa rona, leswaku ri kuceteriwa njhani hi tindzimi tin’wana, na ku hlohlotela njhani matheme man’wana. Mahungu lama hlengeletiweke kusuka eka vanyiki va mahungu ma xopaxopiwile hi ku tirhisa tinhlamulo to suka eka vavulavuri va Xitsonga, lavan’wana va vona ku nga Vativinkulu va ririmi naswona lava tirheke etiyunivhesiti na swiyenge swin’wana swa mfumo eAfrika-Dzonga.

## Tidyondzo ta nkarhi lowu nga hundza ta mbulavulo wa makhombo

Hambiloko ku vile na tidyondzo leti valangeke ntirho wa tindzimi eka swa mbulavulo wa timhangu, leswi swi hleriwile hi tihlo ra vavulavuri va tindzimi ta vuhlanganisi, laha to tala ta tidyondzo leti, swi nga endliwa eka matiko lama hluvukeke yo fana na Nhlangano wa Matiko ya Amerika (USA) na Japani, ku nga laha timhangu ti nga tolovela ku endleka kona (Ueseka & Matthewman 2023). Ematikweni lama hluvukaka yo fana na Afrika-Dzonga, ku na tidyondzo titsongo, loko kuriku tikona, ngopfungopfu leti nga valangangiki ntirho wa tindzimixikaya hi nxungeto wa mhangu kumbe malawulelo ya mbulavulo, kambe ku ri leyi vumbeke ndzavisiso hi ku tirhisa Xitsonga, ku nga rin’wana ra tindzimi leti tshikeleriweke eAfrika-Dzonga. Timhaka ta sweswi ta mhangu na tinhlamulo ta mikunguhato ya xihatla a swi koti ku andlala swilaveko swo pimeka swa miganga leyi nga riki ya Xinghezi. Mpfilungano hi ku hambana ka tindzimi na mindhavuko swi vangela xiphiri xa matimba eka vakhumbeki na lava hungu ri fanekeleke ku fikelela vona.

### Mbangu wa ririmi

Ririmi ri andlalekile ku fana ni mihlohlotelo leyi kanganyisaka mahanyelo ya vanhu, swi nga leswi swi na nkoka ku dyondza ririmi eka mbangu lowu katsaka mavonelo ya muvulavuri, swilaveko, na vutivi bya ndhavuko (Miller 1963). Mbangu ku kongomisiwa eka nkatsakanyo wa swiyimo leswi nga kanganyisaka mahanyelo, naswona eka swa ririmi. Mbangu swi yimela ndzunghiso wa swiphemu swa milomo leswi nga na engetelo eka nhlamuselo, leswaha vitaniwaka xivumbeko xa ntivovuvulavuri bya ririmi (Miller 2007). Ku nga ri hi ku van’wana va titwa njhani hi swivumbeko swa ririmi na ndlela leyi swi nga va olovelaka ha yona, swi nga ha va na mpfilungano kumbe swi nga twisiseki eka van’wana. Xikombiso xa Xikhoriya: ‘Ndzilo nakambe yindlu wu sungurile’, leswi nga ta ka swi nga twisiseki eka vavulavuri va Xinghezi, hambiloko marito lawa ya ri na nandzelelano wa kona hi Xikhoriya (Abukhalaf & Von Meding 2021).

### Xitalo xa matirhiselo ya Xinghezi eka swa mbulavulo

Ririmi i xitirhisiwa xa nkoka eka swa mbulavulo wa xihatla (O’Brien & Federici 2019). Mitlawatsongo leyi tshikeleriweke a yi tali ku katsiwa eka nkunguhato wa xihatla eka swiyenge swa tidyondzo ta le henhla (Méndez, Flores-Haro & Zucker 2020). EAfrika-Dzonga, vavulavuri va Xinghezi Ririmi ro Sungula eka tinxaka hinkwato va kwalomu ka mamiliyoni ya 3.5, kambe Xinghezi xi tekiwa ku ri xona xa le ‘xikarhi’, xo kala mbala, kambe ku nga ri hi ndhavuko kumbe ririmi ra vaaki (Silva 1997). Ku engetela kwalaho, Xinghezi xi hlayisiwa hi Vantima va Afrika-Dzonga lavo tala tanihi ririmi ra ntshuxeko leri kondletelaka vun’we eka MaAfrika-Dzonga hi ku hambana. Hikwalaho, swi tlhela swi va swi olovile ku va ri amukeriwa ku ta hlawulekisa tiko. Tidyondzo to tala ti pfumelelana na ntiyiso wa leswaku tindzimixikaya ta tikokulu Afrika ti tlhontlhiwile naswona ti ya emahlweni no tlhontlhiwa hi Xinghezi. Hikuya hi De Klerk (ed. 1996), Xitsonga, ku fana na tindzimi tin’wana, xi tshikelelekile hikwalaho ka Xinghezi lexi tekiwaka tanihi ririmi ra le xikarhi naswona ra xiyimo xa le henhla eAfrika-Dzonga. Leswi swi vangiwa hikuva Xinghezi xi tirhisiwa tanihi ririmi ra tipolitiki, dyondzo, na ikhonomi. Hi tlhelo rin’wana, ku na ku hungutiwa ka matimba na ku khiriwa ka tindzimi tin’wana. Swiritano, leswi swi hlamusela leswaku vavulavuri vatsongo va tindzimi ta xintu lava nga riki na nkalankanghamo eka tindzimi ta tiko ta ximfumo va le ka xiyimo xa ntsandziso tikweni, tanihi le matikweni yo tala hikuva mbulavulo wa khombo wu hundzisiwa hi tindzimi ta vuhlanganisi leti laha Afrika-Dzonga ti katsaka na Xizulu na Xisuthu. Hambiloko Vumbiwa ra Afrika-Dzonga (1996) ri tlakusile tindzimi ta xintu, ku katsa na Xitsonga tanihi rin’wana ra tindzimi ta ximfumo, Xinghezi xi tama xa ha tiphina swinene hi xiyimo xa ximfumo ku tlula tindzimi letin’wana (Phakeng 2019). Xinghezi xi ya emahlweni xi tirhisiwa tanihi ririmi ra vufambisi, goza leri tiyisisaka ku tshemba ka leswaku mfumo wu hava xikongomelo xa tipolitiki xo hlohlotela ku tirhisiwa ka tindzimi ta xintu. Leswi swi na xiave xo biha eka mbulavulo wa khombo hambiloko swi fanerile. Ku fana na vukondleteri bya NDMC, mbulavulo wa mhangu wu fanerile ku tirhisa tindzimi to hambana etikweni tanihi leswi ntivotindziminyingi wu byarhiwile eka Vumbiwa. Vutivi bya misava byi komba leswaku ku khiriwa ka ku tirhisiwa ka tindzimi ta xintu swi yisa eka ku lahlekeriwa ka vutomi eka swiyimo swa mbulavulo wa swa timhangu tanihi leswi ku nga riki un’wana na un’wana eka ntlawa lowutsongo wa vanhu wu twisisaka ririmi ra vuhlanganisi. Xikombiso, Uekusa (2019) u vike leswaku xikhiro xa tindzimi letitsongo eka swa mbulavulo wa makhombo swi endlile leswaku ku va na ku lahlekeriwa ka tinhundzu na vutomi eJapanivuxa hi nkarhi wa ndzhinginiko wa misava lowukulu eJapanivuxa na Tsunami wa 2011 (lowu tivekaka na hi vito ra khomboxitshuketa ra Tohoku). Endzhaku, ntalomatirhiselo ya ririmi ya nga vangela ku lahlekeriwa ka tindzimixikaya, naswona swiendleko swi lemukiwile ku humelela na hi swidyondzeki swo fana na De Klerk (ed. 1996:7) loyi a boxaka leswaku *ku na xiendleko xa ntiyiso xa leswaku vutihloniphisi, vulawuri, na vululami bya swavanhu, kun’we na ku lahlekeriwa ka ririmi ra munhu, swi nga va na mbuyelo kusuka ka hangalako wa Xinghezi, naswona mhaka leyi i ntiyiso na hi Afrika-Dzonga*.

## Tinhlamuselo ta matheme

### Mhangu

Hikuya hi Nawu wa Afrika-Dzonga, *mhangu* kumbe ‘disaster’ swi vula ku humelela, xitshuketa, hangalako, kumbe vundhawu. Mhangu yi nga va ya ntumbuluko kumbe yo humelela hi ku vangiwa hi munhu leyi (a) vangelaka kumbe ku xungeta (i) rifu, mbanga, kumbe vuvabyi; (ii) ku onha nhundzu, muako, kumbe mbangu; kumbe ku onha vutomi bya muganga (Disaster Management Act No 57 of 2002).

### Swaxihatla

Hikuya hi Shen and Shaw (2004:2110), ‘emergency’ kumbe *swaxihatla* ku kongomisiwa eka xiyimo xin’wana ni xin’wana xa ntumbuluko kumbe xo endliwa hi munhu lexi nga helelaka ka ku onhaka lokukulu, ku nga ha va eka vanhu kumbe nhundzu. Swaxihatla xi nga ha hlamuseriwa tanihi xiendleko lexikulu ngopfu, lexi xungetaka nhundzu, vanhu, kumbe mbangu, naswona lexi lavaka nhlamulo ya nkondletelo na xihatla.

### Nkayakayo

Nkayakayo kumbe ‘crisis’ swi hlamuseriwa tanihi xiyimo lexi nga riki kahle, xi tisaka nxungeto eka vanhu na mabindzu, naswona xi nga vangela ku cinciwa ka pholisi ya xihatla eka munhu un’wana na un’wana (Sawalha, Luai & Kamal 2013). Nkayakayo i ku onha loku khumbaka sisiteme ya xintumbuluko hinkwayo na ku xungeta tshaku ra byarho, ku twisisa mhaka ya yona hiyoxe, ni vukona bya yona (Pauchant & Mitroff 1992). Hi mavonelo ma Booth (1993), nkayakayo i xiyimo lexi munhu, ntlawa, kumbe nhlangano swi hlanganaka na wona; ku nga lexi swi nga koteki ku fambelana na xona hi ku tirhisa maendlelo ya kahle ya ntolovelo, naswona lexi ntshikelelo wu vangiwaka hi ku cinca ka xihatla.

### Nxungeto na Nghozi

Matheme ya ‘risk’ na ‘hazards’ ma Xinghezi ma hlamuseriwa ku hambana. Hikuya hi The Economic Times (2023), *nxungeto* swi komba nchumu wo ka wu nga tshembekangi mayelana na ku hambana kusuka ka mbuyelo lowu languteriweke. Leswi swi hlamuseriwile eka swivulwa. Vuxokoxoko byitsongo byi kumiwile eka mahungu lama nga hansi ka nhlamuselo.

### Nxungeto na nkalovuhlayiseki

Hikuya hi SAMHSA (2017), *nkavuhlayiseki* hi xitalo swi kongomana na nxungeto lowukulu wa ntokoto wo homboloka, switandzhaku, na swiangulo swa khale, swa sweswi, na swa le ndzhaku ka khombo, leswi nga na vuswikoti byo papalata ku hlamula eka switiviso swa khombo, na ku va swi kota ku fikelela mpfuneto lowu landzelaka endzhaku ka khombo.

## Rimba ra thiyori

Dyondzo leyi yi leteriwa hi thiyori ya Bourdieu naswona yi nyika tshaku ra thiyori ya nkanelo wo hlela na xivumbeko eka tindziminyingi tanihi mpfanganiso wa *qhinga ro hunguta nxungeto wa makhombo* (DDR hi Xinghezi). Nsusumeto lowukulu wa thiyori ya Bourdieu hileswaku *qhinga ro hunguta nxungeto wa makhombo* wu kongomanile na matirhiselo ya ririmi leri tirhisiwaka ngopfu, leri nga rihika eka vatshikeleriwa leswi yisaka na le ku hambaneni na vutomi na swiyimo swo byi kanganyisa. Leswi i ntiyiso, ku nyikiwa vumbhoni byo enela bya mhangu kumbe mahungu ya xihatla swi hundzisiwa ntsena hi ririmi/tindzimi leti tirhisiwaka ngopfu. Lava va nga fikeleriki eka tindzimi leti tirhisiwaka ngopfu eka mimbulavulo ya makhombo erixakeni va kumeka ku twisisa ka vona ku tsan’wiwa eka mahungu lama fambelanaka na khombo. Ku fana ni swoleswo, atikili leyi yi kongomana na ku tlakusa matirhiselo ya tindzimi ta xintu to fana na Xitsonga, ku nga ririmi rin’wana ra leti tshikeleriweke eAfrika-Dzonga (Ueseka & Matthewman 2023).

## Mikumisiso na nkanelo

### Khombo

Leswi nga kumiwa kusuka ka nhlokohliso wa swivutiso lowu kunguhatiweke swi hambanile hi ku landza tindhawu timbirhi eka swifundzhakulu swimbirhi laha ririmi leri lavisisiwaka ri vulavuriwaka ngopfu kona, ku nga eGiyani (Xifundzhakulu xa Limpopo) na Bushbuckridge (Xifundzhakulu xa Mpumalanga). Nhlamuselo yo fana ni leyi kumiweke kusuka ka Vanyiki va mahungu hinkwavo vo huma Giyani A, C, na D, ya *nxungeto* kumbe *Nghozi*, yi hambanile na ya Munyiki wa mahungu A, loyi a humaka eBushbuckridge. ‘Disaster’ i *Nghozi* kumbe *Tshumba*. Ku hambana na hinkwavo, Munyiki wa mahungu C, loyi na yena a humaka Giyani, u nyikile nhlamuselo ya theme kumbe rito leri hi ku ri hlamusela kunene leswaku i ‘xiyimo lexi nga vavisaka no hlundzukisa vanhu’. Ku hambana ka vavulavuri lava tirhisaka ririmi rin’we ku hi komba leswaku a ku na nhlamuselo yin’we leyi amukeriwaka yi ri yoxe, leswi pfumelelanaka na Shaluf et al. (2003), hambiloko emasunguleni swi katsaka swivumbeko swo fana. Ku hambana hi nhlamuselo ya *nxungeto* exikarhi ka tindhawu leti swi nga va swi vangiwa hi matshamelo, ikhonomi, na xiyimo xa tipolitiki ta Afrika-Dzonga, tanihi laha swi kombisiweke hi Eshghi and Larson (2008) ku va swa nkoka ku vangela ku hambana loku. Leswi swi tala ku va na ntiyiso hikuva Bushbuckridge yi kwalomu ka ku tlula madzanambirhi ma tikhilomitara kusuka eGiyani, ku vandzakana na Mozambique, naswona ku na Vavulavuri va Xichangana vo huma Mozambique, lexi tirhisiwaka ku vula Xitsonga. Hikwalaho ka vutshamo bya kwalomu ka malembe ya ntlhanu, mutsari wo sungula u vile mbhoni ya ku vona ku hambana hi yexe. Leswi swi vula leswaku loko xilemukiso xa *Nxungeto* xi tirhisa rito ra *Tshumba*, vavulavuri va Xitsonga vo huma eGiyani a va nge ri twisisi hungu ra kona handle ka loko vo va va tshamile kwale nkarhi wo leha. Leswi swa fana na lava humaka eBushbuckridge loko ko tshuka ku ve na mhangu eGiyani loko swi ta eka rito *Nxungeto*. Ku fanana ka tinhlamuselo hinkwato ta *Nxungeto* kusuka eka swidyondzeki, nhlamuselo ya *Nxungeto* yi na swihlawulekisi swa ku ka swi nga vhumbeki, ku lahlekeriwa hi vutomi na nhundzu, ku pfumala matimba ya ku swi lulamisa eka xiphemu lexi langutaneke na swona, ku fana na ndyangu, muganga, na mfumo. Leswi marito *Khombo* na *Nghozi* ma tirhisiwaka hi ku cincana, ma tlhela ma va na ndhuma eka vavulavuri lava nga riki rixaka ra Vatsonga. Leswi swi nga ha bumabumeleka hi mifungho leyo tsariwa ya Xinghezi, kambe leyi hundzuluxeriweke eka tindzimi to hambana etindhawini to fana na switichi swa gezi, ku vona leswaku vanhu va kuma hungu hi xitalo ku fambela ekule na swona. Hi nkarhi wo hlaya, vavulavuri lava nga riki Vatsonga na vona va fikelela ku hlaya nhlamuselo leyi hundzuluxiweke, ku fana na ya marito ya Xitsonga *Khombo* na *Nghozi*. Mianakanyo ya vumbirhi hileswaku *Khombo* ri tirhisiwa na le ka Xivhenda, loko *Nghozi* ri fambelana na ‘Ingozi’ ra Xizulu ro vula *Nghozi*. Swi tlhela swi va erivaleni kusuka eka ku fanana na ku yelana ka matheme lama leswaku ku na ku amukela na ku tekelela ka metheme ya *Nghozi*, tanihi leswi swi nga toloveleka eka tindzimi, ngopfungopfu eka matheme ya Xinghezi. Hi nkarhi wolowo, ku fanana ka marito swi vula leswaku mbulavulo wa mhangu leyi fambelanaka na marito yo tano wu nga ha fikelela nhlayo yo tala ya vanhu handle ka xilaveko xa vuhundzuluxi eka xiyimo xin’wana na xin’wana laha ku nga na vanhu vo hambana hi tinxaka.

### Swaxihatla

Leswi nga kumiwa hi valavisisi va dyondzo leyi mayelana na theme ra ‘emegerncy’ swi fana swinene na leswi boxiweke hi Shen and Shaw (2004). Hikuya hi Munyiki wa mahungu A, ‘emergency’ swi vula ‘swaxihatla’, leswi vulaka ‘xiyimo xa xihatla’. Hikuya hi Munyiki wa mahungu C, swaxihatla swi vula ‘xiyimo xa xihatla lexi lavaka ku tekeriwa enhlokweni xi nga si lahlekela munhu xi hundzuka nghozi. Xiyimo lexi xi va xi nga kunguhatiwangi, kambe xo endleka swi nga languteriwangi’. Hambiswiritano, loko ku tirhisiwa rito ra Xitsonga *swaxihatla* kumbe *ku na xiyimo xa xihatla* eka swa mbulavulo wa nghozi, ku na ku twanana ka leswaku rito leri a ri na ndhuma tanihi leswi ri nga tirhisiweki ngopfu, ngopfungopfu hi vantshwa. Ematshan’weni, matheme ya Xinghezi hi wona ma tirhisiwaka. ‘Swi twakarisa swin’wana ku tirhisa ririmi ra manana eka swiyimo swa xihatla. Ndzi vona onge hi le ku hundzukeni ka lavo basa ku nga ri Vantima’, ku boxa Munyiki wa mahungu C. Hinkwako, vanhu va swi veka hi ndlela leyi loko *swaxihatla* swi humelela: ‘ku na ‘emergency’ la, leswi kombaka leswaku leyi hi yona nhlamuselo leyi tirhisiwaka hi vanhu vo tala. Theme ra *Nghozi* na rona ra tirhisiwa tanihi rito ro siva *swaxihatla.* Hikuya hi Munyiki wa mahungu E: ‘Mikarhi yin’wana ndzi tirhisa “danger” ematshan’wini ya swaxihatla’. Leswi swi komba leswaku marito *swaxihatla* na *nghozi* ma tirhisiwa hi ku cincana tanihi leswi ma nga kumiwa ku fana eka mahungu.

### Nkayakayo/swiphiqo

Hikuya hi Munyiki wa mahungu A, ‘crisis’ swi vula ‘xiyimo lexi nga lahlekeriwa hi vulawuri lexi nga yisaka nghozini’. Mhaka ya leswaku nkayakayo wu nga vangela *nghozi* swi komba vuxaka exikarhi ka matheme yo fambelana na *nghozi*, lama tlhelaka ma nyikiwa eka nhlamuselo ya Munyiki wa mahungu C. Swiphiqo kumbe nkayakayo swi nga vula *nghozi* na *swaxihatla*. Loko munhu a ku ku na *swiphiqo* kumbe *khombo*, swi fana na loko a ku ku na *nghozi.* Munyiki wa mahungu C u boxa leswaku ‘i xiyimo lexi lavaka ku hatliseriwa kumbe ku lulamisiwa’. Ntiyiso wa leswaku vatsari va huma eka Swifundzhatsongo swo hambana swa Xifundzhakulu xa Limpopo swi komba leswaku ncincano eka matirhiselo ya khombo, nghozi, na swaxihatla a swi pimeriwangi xifundzha xin’we, kambe swi kona eka swifundzha hinkwaswo. Munyiki wa mahungu D, u hlamurile hi ndlela leyi:

A ndzi na ntiyiso kahle leswaku ndzi nga ku ‘crisis’ swi vula yini. Wo ndzi kahlula ndzi yi kamba eka dikixinari ya Tsonga/English. Ndzi ehleketa ku vanhu va langutana ngopfu na Xinghezi ku tlula tindzimi ta vona hikuva Xinghezi xi tekeriwa ehenhla naswona xi tirhisiwa eka mabindzu, swikolo, nawu, na swin’wana. Hi tshamela ro ehleketa leswaku hi tiva Xitsonga kasi a hi swona. Ku vula ntiyiso eka wena, a ndzi nge swi koti ku vula leswaku ‘crisis’ swi vula yini hi Xitsonga, naswona ndza tisola eka leswi. Xinghezi xi tirhisiwa kun’wana na kun’wana, ximfumo, na swo ka swi nga ri swa swifumo, naswona eka mburisano njhe, hi tirhisa ngopfu Xinghezi leswi hi katsongotsongo hi le ku dlayeni ka ririmi ra hina. Hi twa onge un’wana na un’wana wa xi tiva leswi ku nga riki swona, ngopfungopfu lavakulu. (Tluka ra khume)

Ntshaho wa Munyiki wa mahungu D, laha henhla, wu hi nyika xifaniso xo andlaleka xa ku lahleka ka matirhiselo ya ririmi ra Xitsonga, ntsena eka mbulavulo wa makhombo. Ntshaho wu nyika vumbhoni bya leswaku vavulavuri va xintu va Vatsonga, ngopfungopfu swidyondzeki na vantshwa, va tirhisa ngopfu Xinghezi, leswi kombaka leswaku Xinghezi xi tekeriwa ehenhla ku tlula ririmi ra manana. Hambiswiritano, swi na vumbhoni leswaku matitwelo ma muxaka lowu hi Xinghezi ma tisa ku tsan’wiwa ka ririmi ra Xitsonga hi venyi va rona, leswi hi ku famba ka nkarhi, swi nga ta endla leswaku Xinghezi xi ya emahlweni xi tirhisiwa ngopfu. Ku tlakuka ka matirhiselo ya Xinghezi swi vula leswaku ku tava na marito mo tala ma xona lama nga ta amukeriwa no ma tekelela ku tlula ma xintu, naswona ku nga ri eka ma ririmi ra vulawuri bya tinghozi ntsena. Xikombiso: ‘emergency’ ri nga amukeriwa ri va *emejesi*, na ra ‘crisis’ ri va *khirayisisi*. Leswi swi tava na switandzhaku swo homboloka eka mbulavulo wa timhangu ta khombo tanihi leswi marito mo talatala ma Xitsonga ma nga ha siviwaka hi lamo amukeriwa no tekelela eka vantshwa na swidyondzeki.

### Nxungeto na khombo

Hikuya hi Munyiki wa mahungu C, ‘risk’ swi vula ‘swilo kumbe swiendleko leswi nga tisaka makhombo’. Hikuya hi Munyiki wa mahungu, ‘risk’ swi tlhela swi vula *khombo* (disaster). ‘Hi ku engetela, “risk” swi tlhela swi vula *nxungeto*’. Hi ku yelanisa, nhlamuselo yo fana yi nyikiwile na hi Munyiki wa mahungu B loyi a kumekaka eka ndhawu yo hambana na ya wa C. *Nxungeto* wu nga va na nhlamuselo yo fana na ya *nghozi* naswona matheme lawa ma nga tirha hi ku cincana. Hinkwaswo leswi swi komba leswaku marito ma nga ha tirhiseka ku olova ku vula *khombo*, naswona leswi swi hi xiswona eka swifundzhakulu leswimbirhi laha Vatsonga va kumekaka kona eAfrika-Dzonga. Hikuya hi Munyiki wa mahungu A, ‘hazard’ swi fana na rito *nghozi*. Loko Munyiki wa mahungu A a nyika xikombiso u te: ‘Ndzi tshamela ro vula leswaku ku na nghozi ekaya loko un’wana a hundzile emisaveni … Ndzi nga ha tirhisa rito khombo ematshan’weni ya nghozi’. Nhlamuselo leyi yi komba leswaku marito mo fambelana na *khombo* ma tirhisiwa hi ncincano eka vanhu naswona ma tirhelana.

### Nxungeto na nkavuhlayiseki

Hikuya hi Vanyiki va mahungu A na C, ‘vulnerability’ swi vula levhele yo tiveka enghozini. Munyiki wa mahungu A, u hlamusela hi ndlela leyi landzelaka:

A ndzi na ntiyiso leswaku swi vula yini hi Xitsonga. Ndzi swi twisisa kahle hi Xinghezi. Ndzi swi lemuka sweswi leswaku a ndzi nge swi koti ku ringeta ku swi hlamusela hi ririmi ra manana. Swa ndzi vilerisanyana loko ku ve hi nga va na rona rito leri vulaka nxungeto hi Xitsonga. Hi vutivi bya mina, a ndzi tshembi leswaku hi na rona. Loko swo lava leswaku ndzi ri hlamula, ndzi nga swi hlamusela kahle hi Xinghezi.

Leswi nga kumiwa laha henhla swi komba leswaku man’wana ma marito ma ntolovelo yo vula *khombo* ya le ku lahlekeni naswona vavulavuri va xintu va le ku ma rivaleni hi katsongokatsongo tanihi leswi va tirhisaka Xinghezi lexi hikuya hi nkarhi, swi nga vangelaka leswaku marito malamo ma nyamalala eka vavulavuri va ririmi. Hikuya hi va Huvo ya misava ya UN, *ku ponisa* i vuswikoti bya sisiteme, muganga, kumbe rixaka leri tikombisaka eka tinghozi, nwelela, hlayisa, tekelela, hundzula, na ku vuyelela eka switandzhaku swa khombo hi nkarhi ni mukhuva wa kahle, ku katsa ni ndlela yo hlayisa no vuyerisela swivumbeko swa masungulo na mitirho hi ndlela ya vulawuri (UNDRR 2017). Swo fanana hi tinhlamuselo ta Vanyiki va mahungu i vuswikoti bya xiyimo ku vuyela eka xiyimo xa khale endzhaku ka xionho.

## Nkanelo wo angarhela

Ndzavisiso lowu wu kumile leswaku hambiloko ku ri na ku hambana ka tinhlamuselo ta matheme ya *makhombo*, nhlamuselo leyi nga exikarhi eka hinkwato hileswaku hinkwawo ma na swihlawulekisi swo yelana swa khombo ku nga: *swaxihatla hi ntumbuluko, swi nga lemukiwangi, swi nga vangaka ku lahlekeriwa na ku onhakeriwa, vutalo,* na *ku katsa swiyenge swa vaaki vo hambanahambana*. Hikwalaho, nhlamuselo ya fana, hambileswi yi nga hlamuseriwaka hi tindlela to hambana. Xin’wana xa swirhangana hileswaku Vatsonga vo tala va tshembela ngopfu eka Xinghezi ku burisana. Ndhuma ya Xinghezi hileswaku xi tirhisiwa tanihi ririmi ra ximfumo etikweni naswona matimba ya rona ma le henhla swinene. Khombo ra kona, tanihi laha swi kumiweke hakona eka mikumisiso leyi kumiweke eka vatirhi eka nhlokohliso wa swivutiso, vatirhi vo hlaya va le ku tsan’weni ka ku tirhisa ririmi ra le kaya hi swintsongontsongo eka mimbulavulo ya khombo, naswona leswi swi nga ha nyanyiseka loko vuahluri byi nga endliwi tanihi leswi vana va vona va nga ha yaka va ya dyondza eka swikolo swa tinxakanyingi. Leswi swi fambelana na mikumisiso ya Neethling (2010) ya leswaku loko vanhu va ya va karhi va fuma, Xinghezi na xona xi nyanya ku navetisa. Swingaleswi, leswi swi va na xiave xo biha eka tindzimi ta Afrika naswona swi ta ya emahlweni swi va tano. Hi ku engetela, loko ku ri na lava kotaka ku vulavula Xitsonga handle ka nkanakano, mikumisiso yi komba leswaku lava nga twisiseki Xinghezi, ngopfungopfu vadyuhari, va ta sala endzhaku loko swi ta eka ku kuma mahungu, ngopfu lama fambelanaka na mimbulavulo yo fambelana na makhombo, ku fana na switiviso swa masungulo leswi burisaniwaka hi Xinghezi. Swoleswo swi nga ha engetela nhlayo ya vavaviseki hi swa makhombo eka vadyuhari emigangeni. Xikombiso, eUSA vanhu va 30 va lovile hi 1987, vo tala lava ku nga vaaki va le Mexico, laha F4 thonado yi nga wela Saragosa, Texas. Rifu ra vona ri hlohloteriwile hi mpfumaleko wa vanhu eka vutivi bya vuhundzuluxeri bya ririmi ra Xipenixi, hikuva xitiviso xa nghozi xi rhumeriwile hi Xinghezi (Benavides & Arlikatti 2010). Leswi swi komba leswaku swi na xiphiqo ngopfu loko vavulavuri va ririmi ra lavatsongo va nga twisisi switiviso/swiviko swa makhombo, naswona loko va nga koti ku burisana na valawuri na van’wana hi nkarhi wa swiyimo swaxihatla. Ku fanana na swona, eAfrika-Dzonga, ku nga ri vuhundzuluxi ntsena leswi endlekaka, kambe hi ku angarhela ku na nkayivelo wa switirhisiwa leswi tsariweke hi Xitsonga, hi ku kongomisa eka mpfumaleko wa switirhisiwa na ku tsakela tanihi xihingakanyi lexikulu lexi endlaka leswaku vanhu lavatsongo na lava nga dyondzekangiki va nga vi eka xiyimo xo hlayiseka. Tanihi laha swi kombisiweke hi IFRC (2018), leswi swi na switandzhaku swo hundzisa hungu, loko swi koteka, a swi katsiwi eka tipulani to tlakusa ku ponisa, kambe ku pfumaleka ka swona swi tlakusa switandzahaku swa swirilo swo hundzisa mahungu. Onge leswi kayivelaka hinkwako laha tikweni i maqhinga yo hluvukisa mbulavulo wa makhombo eka tindziminyingi.

## Swibumabumelo

Mbulavulo mayelana na mindzindzakhombo eka tindzimi to hambanahambana wu na nkoka ku antswisa tilevhele to tilulamisela eka tinxaka leti nga sirhelekangiki, ku katsa na tinxaka hi mbala ni muhlovo. Loko tipholisi na milawu eAfrika-Dzonga swi nyika mbulavulo wa mindzindzakhombo hi tindzimi hinkwato, tindzimi letitsongo to fana na Xitsonga a tikumi vukorhokeri, ku karhi ku kongomisiwa eka mpfumaleko wa switirhisiwa. Hikuya hi mitiyiso ya sweswi, loko ko ka ku nga endliwi nchumu ku tlakusa tindzimi ta xintu, vavulavuri va tindzimi ta xintu va ta amukela no tekelela marito mo tala ma Xinghezi leswi nga yisaka eka ku lahlekeriwa hi matheme ya xintu. Nkateko ‘fly-machine’ leri nga cinceriwa eka ra *Fulayimachini*, swi vula leswaku ro fana na ‘emergency’ ri nga tekelela ri va *emejesi*, na ‘crisis’ ri va *khirayisisi*. Ku hlayisa na ku tekelela ka marito ku tolovelekile (Neethling 2010), kambe swi fanele swi humelela ku nga ri eka marito ku hlamusela mhaka yo karhi hi ririmi ro tekela. Vuswikoti byo vulavula Xinghezi a byi vuli ngopfu leswaku Vatsonga va fanele ku tsan’wa ririmi ra le kaya, kambe va fanele ku ri hlayisa eka swa mbulavulo wa vona, ngopfungopfu kusukela loko swi twisisiwile hi vanhu hinkwavo, ku katsa na vadyuhari, lava ku nga vona ngopfu va nga eka xiyimo xa mpfumaleko wa nhlayiseko. Hambileswi swi tsundzuxekaka ku va n’watindziminyingi, vutivi bya tindziminyingi a byi fanelangi ku humelela hi ku tshkilela tindzimi tin’wana ta xintu; ngopfungopfu letitsongo to fana na Xitsonga, ku nga leti nga na ndhuma yintsongo. Hi ku engetela eka ndhavuko na tindlela leti lavekaka ta mbulavulo wa makhombo (xik.: makombakule, xigilamukhuva, vatirhi va le handle, mitirho ya un’wana na un’wana), swi tlhela swi va na nkoka ku hlohlotela vufanisi loko ku tirhisiwa inthanete na switirhisiwa swa ximanguvalawa swa thekinoloji loko ku avelaniwa mahungu. Leswi swi ta endla leswaku tindlela to hambana ti tirhisiwa, tanihi leswi mitlawa ya vanhu yi hambaneke hi ku landza mikhuva lowu tirhisiwaka eka mbulavulo. Hambiswiritano, ku yima na Garnett and Kouzmin (2009), hambiloko swi nga yi hi tindlela kumbe mukhuva, ku avelana mahungu swi nga ha tirhiseka hi ku tirhisa Xitsonga tanihi ririmi ra le kaya eka swiyimo swa mbulavulo wa ndzindzakhombo tanihi leswi swi boxaka mhaka, naswona loko ku avelaniwa na vavulavuri va tindzimi tin’wana hi nkarhi wa kona. Thekinoloji a ku ri ndlela yin’wana leyi tirheke hi nkarhi wo angula Katrina. Vaaki va rhumerile mahungu ku ya eka un’wana hi ku tirhisa tiqinghonyonga. Hikwalaho, maqhinga yo byarha mbulavulo wa ndzindzakhombo a wu fanelangi ku languta ntsena tindlela ta ntolovelo na leti languteriwaka (xik.: makombakule, xigilamukhuva, vatirhi va le handle, mitirho ya un’wana na un’wana), kambe ku engeteriwa hi inthanete na switirhisiwa swa ximanguvalawa swa thekinoloji, ku endlela leswaku tindlela to hambana ti va kona na ku pakanisa vaaki kusuka eka mitlawa ya vanhu vo hambana na swilanguteriwa ku nga leswi pfumelelanaka na Garnett and Kouzmin (2009). Mahungu ma nga ha hangalaseka hi mihlangano ya muganga leyi nga na vuxaka na mindhavuko yo hambana na tindzimi ta miganga, naswona leswi swi nga endliwa hi ku burisana na varhangeri va mihlangano ya miganga, vafundhisi va tikereke, na varhangeri van’wana va vukhongeri, vo fana na vaprista epedagogweni kumbe etempeleni. Mihlangano ya muxaka wolowo yi nga hangalasa hungu ra ntiyiso hi ndlela ya kahle eka levhele ya xikaya.

## Nghimeto

Ndzavisiso lowu wu xoperile nhlamuselo ya matheme mo hambana lama tirhisiweke eka Xitsonga na ndlela leyi ma hlohloteriwaka hakona hi tindzimi tin’wana etikweni na swifundzhakulu. Vuxokoxoko hinkwabyo bya pureyimara na bya sekondari byi tirhisiwile, endzhaku byi hlengeletiwa kusuka eka vatsari va Xitsonga. Ndzavisiso wu kumile leswaku marito kumbe matheme ya vulawuri bya makhombo yo fana na *khombo, nghozi, swaxihatla,* na *nkavuhlayiseko* ma tirhisiwile hi ku cincana kusuka ka mahungu na mikumisiso ya savheyi, na leswaku mbulavulo wa khombo wu hundzisiwa ngopfu hi Xinghezi, leswi endliwaka ehenhleni ka vavulavuri va Xitsonga. Nkumisiso wun’we wa nkoka hileswaku vavulavuri va Xitsonga va xintu va na vutivi byitsongo bya marito yo vula khombo, leswi va swi amukelaka, ku nga leswi va khumbaka tanihi leswi swi nga ha ndlandlamukaka loko ku nga ri na nghenelelo wo sivela ku herisiwa ka ririmi.
